# Does postural sway change in association with manual therapeutic interventions? A review of the literature

**DOI:** 10.1186/2045-709X-21-9

**Published:** 2013-02-04

**Authors:** Alexander Ruhe, René Fejer, Bruce Walker

**Affiliations:** 1 School of Health Professions, Murdoch University, Murdoch, Western Australia, Australia; 2 Praxis fuer Chiropraktik Wolfsburg, Wolfsburg, Germany; 3 Research Department, Spine Centre of Southern Denmark, Hospital Lillebaelt and University of Southern Denmark, Middelfart, Denmark

**Keywords:** Center of pressure, Postural sway, Manual therapy, Intervention

## Abstract

**Study design:**

Literature Review

**Objectives:**

The objective of this literature review was to determine if postural sway changes in association with manual therapeutic interventions and to investigate whether any changes occur in healthy individuals or in association with pain intensity.

**Summary of Background data:**

Improving postural stability has been proposed as a goal of manual therapeutic interventions. So far, no literature review has addressed whether there is supportive evidence for this and if so, what factors may be associated or causative for observed sway alterations.

**Search methods:**

Seven online databases (PubMed, MEDLINE, EMBASE, CINAHL, Web of Science, ScienceDirect and the Cochrane library) were systematically searched followed by a manual search of the retrieved papers.

**Selection criteria:**

Studies comparing postural sway derived from bipedal force plate measurements in association with a manual therapeutic intervention, ideally compared to a control group.

**Data collection and analysis:**

Two reviewers independently screened titles and abstracts for relevance, conducted the data extraction and the risk of bias assessment which was conducted using the RTI item bank. A descriptive analysis was conducted as the heterogeneous study designs prevented pooling of data.

**Results:**

Nine studies of varying methodological quality met the inclusion criteria. No direct comparison of data across the studies was possible. There was no evidence that manual interventions lead to a change in postural sway in healthy individuals regardless of the body regions addressed by the intervention. There was some indication that postural sway may change at follow-up measurements in pain sufferers; however, this may be due to variations in pain intensity rather than resulting from the intervention itself.

**Conclusions:**

There is no conclusive scientific evidence that manual therapeutic interventions may exhibit any immediate or long-term effect on COP excursions. Any changes in sway may be attributable to decreases in pain intensity.

## Background

Restoring postural stability and balance has been advocated as one goal of therapeutic interventions throughout the physical medicine professions [1] and changes in center of pressure (COP) excursions as a measure of balance performance in association with therapeutic exercise [2-4] or balance training [5-7] are well documented in the literature.

After applying spinal manipulation as an intervention, several studies have reported treatment effects on differences in weight distribution between the lower extremities [8] and balance performance by means of the Berg balance scale (BBS) [9-12].

It appears likely that any change in COP excursions associated with manual interventions is due to a reduction in pain perception [13,14]. In addition, it may be speculated that a therapeutic intervention capable of increasing somatosensory function may be beneficial for postural stability. For example, spinal manipulative therapy (SMT) of the cervical spine has been shown to improve proprioception [15,16], although the underlying mechanism(s) remains unclear.

Despite the theoretical neuro-physiological associations between spinal manipulation and postural stability, only a few studies have been published and thus the evidence of the mechanisms of spinal manipulation on COP excursions remains unclear. This literature review will present and critically comment on the current state of knowledge.

The objective of this literature review is to 1) determine if there are significant changes in postural stability associated with manual therapeutic interventions, 2) investigate whether these changes occur in pain sufferers, healthy individuals or both and 3) whether any observed postural sway alterations are related to factors such as pain intensity associated with the underlying condition of the symptomatic individuals.

## Methodology

For the purpose of this review, AR acted as the principal reviewer. A colleague experienced in literature review data extraction was involved independently in the process of identifying relevant studies but did not participate in further analysis of the finally included papers.

### Search strategy

A comprehensive search strategy was developed to identify all potentially relevant studies.

Basic inclusion criteria were those studies investigating postural sway exhibited by symptomatic or asymptomatic individuals on a forceplate following some form of manual therapeutic interventions such as manipulation, mobilization or massage. Studies employing rehabilitative interventions such as proprioceptive training or muscle strengthening exercises only were excluded.

Key indexing terms were categorized into specific search phrases and subsequently combined by using Boolean terms. This search strategy was applied to seven different electronic databases: PubMed, MEDLINE, EMBASE, CINAHL, Web of Science, ScienceDirect and the Cochrane library. The date range of publications searched was from January 1980 to May 2012.

A subsequent hand search was conducted through the reference lists of all the included studies. Citation searches of relevant studies were conducted using the PubMed, MEDLINE and ScienceDirect databases.

This search strategy Initially provided only a limited yield, accordingly the inclusion and exclusion criteria were extended to include any type of publication in order not to miss potentially relevant papers.

### Risk of bias assessment

A risk of bias assessment was conducted independently by two reviewers (AR and RF) in order to determine the quality of the included studies. Recently, Viswanathan et al. have identified 29 practical and validated items that may be used to evaluate the risk of bias and precision of observational studies [17]. This bank of items covers a range of different study designs and the authors have provided instructions as to what items to use depending on the studies under assessment.

Thus, only five items related to our main objectives were included and criteria for each item were defined to fit our main objective (Table 1). The layout of the questionnaire was slightly modified for practical reasons, but no other changes were made. The chosen items focused on selection bias, precision, performance and information bias, and the overall interpretation of each study. Relevant criteria to assist in determining the risk of bias in a study were specified to each item. No validation of the included items was performed.

**Table 1:** RTI Items elected to assess risk of bias and precision of the included studies

Item number from original study*	**Dimension of bias**	**Methods domain**	**Assessment question**	**Criteria / definitions / categories**
2	Selection bias	Sample definition and selection	Are critical inclusion/ exclusion criteria clearly stated?	• Age range, gender, etc. described?
				• Specific inclusion/exclusion criteria stated?
6	Precision	Sample definition and selection	Was the sample size sufficiently large to detect a significant difference between groups?	• Justification for selected sample size given?
				• Were sample size calculations performed?
7	Performance bias	Interventions/exposure outcomes	What is the level of detail in describing the intervention?	• Type of intervention, timing and frequency described?
				• Was the intervention identical for all participants?
9	Selection bias	Creation of treatment groups	Is the selection of the comparison group appropriate	• Is there a comparison/ control group?
				• If so, are there fundamental differences between the groups on the basis of socio-demographic variables and the outcome variables at baseline?
				• Do the controls represent the population from which the intervention group arose?
15	Information bias	Soundness of information	Are the outcomes assessed using reliable measures?	• Was the reliability of the outcome assessment tested?
				• If not, is the measurement protocol likely to yield reliable/valid results with regards to
				- sampling duration
				- number of repetitions
				- visual condition
				- foot position
				based on a systematic review of the literature [18]
			Overall judgment	• Low risk of bias: Bias, if present, is unlikely to alter the results seriously
				• Unclear risk of bias: Impossible to determine risk of bias (either missing or not described well enough)
				• High risk of bias: Bias may alter the results seriously

* [17].

Comparing post-intervention results with baseline values may be deemed sufficient to assess for a treatment effect. However, Question 9 was included in the risk of bias assessment as a comparison group is useful to determine whether changes in the outcome measures were due to the intervention or effects of learning or fatigue due to repetitive testing.

Where authors did not provide information on the reliability of postural sway assessment, a judgment of methodology was made based on a previous systematic review of the literature. For example, three repetitions of 90sec measuring duration in narrow stance (feet together) with eyes closed were deemed appropriate [18].

It was decided that a study with one or more of the key items being rated negative or unclear could not be rated as of low risk of bias.

### Data analysis

A descriptive analysis was conducted as the included studies were significantly heterogeneous with regards to study design, intervention, characteristics of participants and outcome measures (e.g. postural sway parameters and experimental procedures), which prevented any pooling of data.

## Results

### Study selection

**Figure 1:**
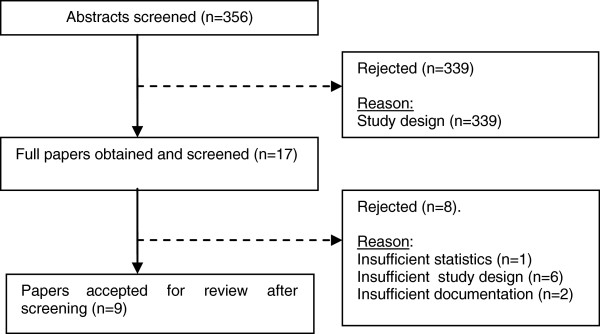
Flowchart of papers.

The second and final database search strategy identified 356 studies of which all abstracts were screened. The application of inclusion and exclusion criteria eliminated 339 papers. From the 17 remaining articles, the full text of the papers was reviewed and 8 more were eliminated leaving 9 studies finally included in this review. Of these, six were published in peer-reviewed journals [19-24], one of them as a single case study [20]. The remaining studies were undergraduate student projects (Figure 1).

### Risk of bias assessment

The risk of bias assessment showed that all studies were of high or unclear risk. None of the included studies provided sample size calculations to investigate when statistically significant differences pre- and post-intervention or between intervention and control groups could be reached.

Only two of the included studies tested the reliability of the experimental setup used and found satisfactory results (ICC≥0.75) [22,23]. For the remaining studies, the expected reliability of the postural sway measures based on methodological recommendations in a previous literature review [18] was low or unclear (Table 2).

**Table 2:** Assessment of risk of bias and precision

**Study**	**Q2**	**Q6**	**Q7**	**Q9**	**Q15**	**Overall judgment on risk of bias**
Persson et al. [21]	+	-	+	-	-	unclear
Lafond et al. [20]	unclear	-	+	-	unclear	high
Jones [27]	-	-	+	-	unclear	unclear
Vaillant et al. [19]	unclear	-	unclear	-	-	high
Nolan [28]	+	-	+	-	unclear	unclear
Ruhe et al. [22]	+	-	+	-	+	unclear
Levy et al. [29]	unclear	-	-	+	+	unclear
Alburguerque-Sendin et al. [23]	unclear	-	+	unclear	unclear	unclear
Giemza et al. [24]	+	-	+	+	-	unclear

+ : yes, - : no.

### Characteristics of participants

While the participant's demographics have been shown to affect postural sway measures [25,26], only half of the studies provided sufficient details on socio-demographic information.

Apart from the case study [20], all other studies used small mixed gender groups of 17 [19] to 42 [27] participants. The mean age ranged from 22.5 (SD 5.7) [27] to 74.5 (SD 9.6) [19] years. With regards to symptomatic participants, two studies enrolled individuals with neck pain [20,21] and one enrolled patients with non-specific low back pain (NSLBP) [22]. Otherwise, healthy individuals were used (Table 3).

**Table 3:** Participant demographics and health status

**Study**	**Participant health status**	**Gender (n)**		**Age**	**Weight**	**Height**
		**Female**	**Male**	**in years**	**in kg**	**in cm**
				**Mean (SD)**	**Mean (SD)**	**Mean (SD)**
Persson et al. [21]	Cx root compression					
	physiotherapy	14	10	47 (8)	75 (16)	171 (2)
	healthy	8	12	45 (9)	75 (12)	177 (11)
Lafond et al. [20]	chronic neck pain	1	0	45	-	-
Jones [27]	healthy	23	19	22.5 (5.7)	-	-
Vaillant et al. [19]	healthy	0	17	74.5 (9.6)	73.2 (12.3)	165.6 (9.3)
Nolan [28]	unclear	12	10	18-45	-	-
Ruhe et al. [22]	NSLBP	21	17	39.8 (10.5)	79.3 (12.4)	178.1 (8.4)
Levy et al. [29]	healthy	unclear(12 total)		20-50	-	-
Alburguerque-Sendin et al. [23]	healthy	23	9	21.9 (3.4)	-	-
Giemza et al. [24]	hip osteoarthritis	0	80	68.5 (3.7)	75.7 (9.4)	169.6 (6.8)
	healthy	0	30	69.3 (3.2)	74.0 (7.5)	172.3 (5.1)

Cx: cervical, NSLBP: non-specific low back pain.

- : not described.

### Characteristics of the interventions

With three exceptions [23,28,29], the studies used combinations of different forms of manual therapeutic and/or exercise interventions.

The majority of studies based their conclusions on a single session with follow-up COP measurements immediately following baseline measurement and intervention [19,23,28-30]. The others used about one week [31] to 3 month follow-up [21]. A comprehensive overview about procedures and results is presented in Table 4.

**Table 4:** COP excursions associated with therapeutic interventions

**Study**	**Intervention**			**Experimental setup**			**Results**			
		**Sampling duration (sec)**	**Follow-up period**	**Number of repetitions**	**COP parameter**	**Postural task**	**Postural sway**		**Pain intensity**	
							**Pre-intervention**	**Post-intervention**	**Pre-intervention**	**Post-intervention**
Persson et al. [21]	Physiotherapy	10	12 weeks	1	mVel (mm/s)	narrow stance				
	Total: 15x e.g. exercise, massage					EO/F	11.4	12.9	47 (8) VAS	39 (29) VAS
						EC/F	15.8	15.6		
Lafond et al. [20] †	Total:	30	8 weeks	1	mVel AP	narrow stance			60 (VAS)	20 (VAS)
	Spinal manipulation				(mm/s)	EO/F	~10.0	~5.5		
	16x cervical (HVLA)					EC/F	~13.0	~7.5		
	C2/3 level				mVel ML	narrow stance				
	Rehabilitation				(mm/s)	EO/F	~6.0	~2.0		
	16x strengthening					EC/F	~7.5	~2.0		
	16x oculomotor				area (mm^2^)	narrow stance				
	exercise					EO/F	86.0	100.3		
	16x balance					EC/F	-	-		
	exercise									
	16x stretching									
Jones [27]	1x Spinal manipulation	unclear	same day∞	unclear	mVel (mm/s)	normal stance			N/A	N/A
	lumbar (HVLA)					EO/F	4.5 (1.7)	4.3 (1.8)		
	1x Muscle energy					EC/F	6.0 (2.2)	5.4 (3.0)		
	technique					unipedal stance				
	1x Myofascial					EO/F	17.3 (6.1)	17.0 (3.6)		
	technique					EC/F	38.6 (11.5)	35.4 (11.9)		
						tandem stance				
						EO/F	14.7 (5.5)	12.0 (4.4) **		
						EC/F	25.8 (9.6)	21.4 (8.1) ***		
Vaillant et al. [19] †	Mobilization	EO: 4	same day∞	6	displacement AP (mm)	narrow stance			N/A	N/A
	1x ankle/feet	EC: 8		6		EO/F	36	34		
	Massage				displacement ML (mm)	EC/F	62-68	58-62		
	1x ankle/feet					narrow stance				
						EO/F	47	42		
						EC/F	74-88	67-70		
Nolan [28] †	Manipulation	60	same day∞	2	stability index	normal stance			N/A	N/A
	1x cervical (HVLA)					EO/F AP	2.90	2.10		
	C0/1, C1/2 level					EO/F ML	2.55	1.65		
Levy et al. [29]	Instrument-applied manipulation	10	same	1	sway velocity	unclear stance			N/A	N/A
	(Pro*-*Adjuster System)		day∞		(deg/sec)?	EO/F	0.24 (0.11)	0.22 (0.16)		
						EC/F	0.23 (0.14)	0.19 (0.11)		
						EO/C	0.61 (0.19)	0.53 (0.15)		
						EC/C	1.73 (0.49)	1.33 (0.41) *		
Alburguerque-Sendin et al. [23]	Manipulation	60	same	1	area (mm^2^)	unclear stance			N/A	N/A
	1x talocrural joint		day∞		mVel AP/ML	EO/F area	85.5 (122.3)	52.8 (48.1)		
					(mm/s)	EO/F mVel AP	2.5 (0.6)	2.4 (0.5)		
						EO/F mVel ML	3.0 (0.7)	2.9 (0.6)		
Ruhe et al. [22]	Spinal and extremity	90	~1 week	3	mVel AP/ML (mm/s)	narrow stance				
	3x manipulation					EC/F AP	13.2 (2.9)	11.1 (2.3) ***	5.6 (2.0)	2.9 (1.6)
	(HVLA)					EC/F ML	16.0 (2.7)	13.1 (3.0) ***	(NRS-11)	(NRS-11)
	and mobilization									
	3x Soft tissue									
	techniques									
	(e.g. PIR, ART)									
Giemza et al. [24]	Exercise, massage, PIR, cryotherapy, diathermy, laser	20	6 weeks	1		normal stance			"great pain"	unclear
					area (mm^2^) AP/ML	EO/F AP	65.8 (24.2)	14.8 (17.4)**		
					mVel AP/ML (mm/s)	EO/F ML	31.0 (19.3)	13.1 (13.9)**		
						EO/F AP	89.8 (43.3)	44.1 (25.0)**		
						EO/F ML	65.4 (29.9)	33.1 (46.4)**		

† "Same day" refers to a single session consisting of pre-intervention measurement, intervention and post-intervention measurement.

Results are presented as Mean (SD).

Levels of significance compared to baseline: * *p*≤0.05, ** *p*≤0.01, *** *p*≤0.001.

- : not described.

AP: antero-posterior, ART: Active Release Technique, C: compliant (foam) surface, deg/sec: degrees per second, EC: eyes closed, EO: eyes open, F: firm surface, HVLA: high velocity low amplitude, ML: medial-lateral, mVel: mean velocity, N/A: not applicable, PIR: Post-Isometric Relaxation.

### Changes in COP associated with manual therapeutic interventions

In the study by Jones [27], a single osteopathic high velocity, low amplitude (HVLA) manipulation was targeted to the lumbar region between L1 and L5, depending on the physical examination findings. Furthermore, “muscle energy technique” was included and involved three repetitions of seven isometric contractions and soft tissue techniques were applied bilaterally to the lumbar paraspinal musculature for 45sec. While a significant, immediate reduction in post-intervention mean sway velocity (mVel) was noted in tandem stance with both eyes open (*p*=.003) and eyes closed (*p*=.001), no differences were observed in normal or unipedal stance under either visual condition.

Persson et al. [21] applied manual therapies such as massage while excluding SMT for their group of neck pain sufferers. After 15 applications of therapeutic massage to the neck area and exercise sessions over a 3 month period, no significant post-treatment changes in COP sway were identified and no significant reduction in the perceived pain intensity as assessed by VAS occurred.

The intervention program set up by Lafond et al. [20] for their single case study was diverse and involved HVLA manipulation to the cervical spine in combination with different forms of physical rehabilitation and exercise. A significant reduction in postural sway post-intervention was noted for all included parameters. Mean sway velocity, for example, decreased by 44.1% (AP, eyes open) and to 50.5% (ML, eyes open) after 16 interventions over 8 weeks. The reduction in COP excursions was accompanied by a clinically significant decrease in pain perception from VAS 60 to 20.

Vaillant et al. [19] conducted manual mobilizations of the feet in all planes. Before and after the therapeutic manipulation, the healthy participants exhibited very similar COP displacements with eyes open. With eyes closed, a decrease in postural sway was observed particularly in ML direction. However, this difference was non-significant.

Nolan [28] used the Stability Index (SI) to investigate the immediate effect of cervical HVLA manipulation on postural stability in asymptomatic individuals. The SI represents the variance of the force platform displacement in degrees from a level position in all positions. Greater amounts of body movements are associated with increasing SI values [32]. A statistically significant reduction in post-intervention SI magnitude was noted in the intervention group in both AP and ML direction while the results of the placebo group remained fairly constant.

Alburguerque-Sendin et al. [23] did not find that bilateral talocrural joint manipulation changed COP excursions in healthy subjects. They noted a non-significant trend towards small differences between intervention group and controls not receiving an intervention.

When Giemza et al. [24] assessed postural sway in a group of patients with hip osteoarthritis before and after kinesiotherapy (e.g. massage, exercise), they noted a statistically significant sway decrease post-intervention (p<0.01).

A recent study investigated whether changes in pain intensity would result in changes in the magnitude COP excursions [22]. For this purpose, postural sway was measured according to a best practice experimental setup [18] following non-specific chiropractic "usual care" manual therapeutic interventions. The authors did not include a placebo group as no conclusions regarding causality were intended with regards to changes in COP excursions. However, in this study, a statistically significant overall decrease in both sway velocity and area was observed at the third session following the interventions compared to baseline. Where no pain reduction was achieved there was no corresponding change in COP excursions.

Levy et al. [29] enrolled two groups of chiropractic students. The intervention group (n=12) received “instrument-applied manipulation(s)” according to "previous scan findings", while for the controls (n=11) an un-specified sham treatment was applied. Postural sway measures of both groups were obtained before and after. Only during one of the four postural tasks, where COP was measured post-intervention with eyes closed on a foam surface, a significant decrease in postural sway was noted compared to baseline (p<0.05).

## Discussion

Large scale studies investigating changes in COP excursions associated with manual therapy have been announced at scientific conferences but are yet to be published [33]. COP measures have also been specifically suggested as a monitoring tool for chiropractic practice [1]. This, however, is premature. So far only a few studies have been reported and three of these were under-graduate theses [28-30] that have not been additionally published in a peer reviewed journal to this point.

COP measures are used by some practitioners applying manual therapies, so the lack of good quality studies may suggest that publication bias may play a role. This refers to the tendency on the part of authors to submit, as well as the reviewers and editors to accept, manuscripts based on the study findings [34] as the strongest and most positive studies are most likely to be published [35].

Overall, any interpretation of the reported results is severely limited by a lack of detail in the employed study designs that were often not suited to answer the self-defined research question [19,21,24,28,30]. In addition, the included studies were all found to be either at high or unclear risk of bias.

Important limitations were the absence of a comparison or control/placebo group in all but two studies [23,29], the generally small samples sizes and the often low or unclear reliability of the obtained sway data. For example, the majority of studies generally employed rather short sampling durations. The test-retest variability in postural sway that may occur as a result can lead to the false impression of changes in the outcome measure post-intervention. Although Vaillant et al. [19] used six repetitions, the combined sampling duration of 50sec was still fairly short which may have adversely affected the results.

In addition, no sample size calculations were reported to assess when statistical significance may be reached between intervention and control/placebo groups. Where multimodal interventions were used [19-22,24,27], the effectiveness of particular treatments remains unclear. In those instances where changes in postural sway were reported [19,24,30], it was not possible to determine whether the intervention itself or learning effects due to repetitive testing [36] may have been causative.

Furthermore, the fact that most studies performed the follow-up measurements immediately after the intervention [19,23,28-30], no conclusions can be reached whether any observed changes are sustained. None of the studies using follow-ups of several weeks [20,21,24] employed multiple measurements at regular intervals that may have allowed an appreciation of associations between pain and postural sway or learning effects due to repetitive testing.

However, it appears that when healthy participants were tested, generally no significant change in postural sway between COP excursions pre- and post-intervention was noted [19,23,30]. When Nolan reported a significant decrease in postural sway associated with cervical SMT [28] this may be explained by the fact that the Biodex Balance System was the only forceplate used that allowed surface perturbation and a sway degree based COP parameter to be employed.

With regards to the study by Persson et al., there is no conclusive evidence that massage is an effective treatment for cervical nerve root compression [37]. This may at least partly explain why the perceived pain levels did not decrease significantly and, as a correlation between these two factors exists [38], the COP excursions remained similar to pre-intervention stage.

The results reported by Vaillant et al. [19] further indicate that the mobilization intervention either had no immediate effect on postural sway with eyes open, or that any such effect remained undetectable when allowing visual fixation. This is supported by the decrease in COP displacement under visual obstruction.

Finally, the results reported by Giemza et al. [24] warrant some caution. Firstly, the data collected from the thirty healthy controls to be compared to that of the symptomatic patients was not actually reported in the study. Secondly, no symptomatic controls were selected. The 6 weeks interval between the two measurements of questionable reliability does also allow for many factors to influence postural sway, including learning effects. In addition, it was mentioned that the in addition to increasing range of motion, the kinesitherapy also aimed at reducing the "great pain" of the patients. However, no pain levels were recorded pre- and post-intervention that may offer an explanation for changes in postural sway.

Based on the literature available, there is no conclusive evidence that manual therapeutic interventions exhibit any short term effect on body sway, at least in asymptomatic participants, for the COP parameters employed. Other parameters, such as those based on frequency or amplitude, may provide additional insights.

There is weak evidence that a significant decrease in pain perception in symptomatic individuals was associated with decreasing COP excursions [20,22], while at similar pain perception, postural sway remained unchanged [21,22]. Accordingly, it appears likely that the pain reduction itself is responsible for the observed lower postural sway in those experiencing pain relief.

The manual intervention itself on the other hand does not appear to offer any additional biomechanical or neuro-physiological benefit (e.g. by stimulation of joint mechanoreceptors) compared to natural history or the changes in sway observed under analgesic treatment [39]. However, the limitations of the respective included studies do not encourage further hypothesizing about potential underlying mechanisms at this point. At this point, practitioners are discouraged from advertising any effect of manual therapeutic interventions on balance e.g. in fall or injury prevention.

## Conclusion

Due to the heterogeneous study designs there is no conclusive evidence that manual therapeutic interventions exhibits any immediate or long-term effect on COP excursions in healthy individuals. In pain sufferers, any changes in sway may be attributable to a decrease in pain intensity rather than the intervention itself. Given this heterogeneous reporting, further research needs to implement standardized testing protocols, include control groups, obtain larger sample sizes in order to allow for comprehensive inter-study comparisons and involve follow-up testing.

## Competing interests

The authors declare that they have no competing interests.

## Authors’ contributions

AR and RF conducted the literature search and the risk of bias assessment. AR drafted the manuscript and performed the statistical analysis. RF and BW helped with the design of the study and drafting the manuscript. All authors read and approved the final manuscript.
